# The combination of ulinastatin and somatostatin reduces complication rates in acute pancreatitis: a systematic review and meta-analysis of randomized controlled trials

**DOI:** 10.1038/s41598-022-22341-7

**Published:** 2022-10-26

**Authors:** István László Horváth, Stefania Bunduc, Péter Fehérvári, Szilárd Váncsa, Rita Nagy, Gantsetseg Garmaa, Dénes Kleiner, Péter Hegyi, Bálint Erőss, Dezső Csupor

**Affiliations:** 1 Centre for Translational Medicine, Semmelweis University, Üllői út 26, 1085 Budapest, Hungary; 2 University Pharmacy Department of Pharmacy Administration, Hőgyes Endre utca 7-9, 1092 Budapest, Hungary; 3 Institute for Translational Medicine, Medical School, University of Pécs, Szigeti út 12, 7624 Pécs, Hungary; 4 Division of Pancreatic Diseases, Heart and Vascular Center, Semmelweis University, Baross út 22-24, 1085 Budapest, Hungary; 5 János Szentágothai Research Center, University of Pécs, Szigeti út 12, 7624 Pécs, Hungary; 6 Carol Davila University of Medicine and Pharmacy, Dionisie Lupu Street 37, 020021 Bucharest, Romania; 7 Fundeni Clinical Institute, Fundeni Street 258, 022328 Bucharest, Romania; 8 Budapest Department of Biomathematics and Informatics, University of Veterinary Medicine, István utca 2, 1078 Budapest, Hungary; 9 Institute of Translational Medicine, Semmelweis University, Nagyvárad tér 4, 1089 Budapest, Hungary; 10 Institute of Clinical Pharmacy, University of Szeged, Szikra utca 8, 6725 Szeged, Hungary; 11 Heim Pál National Pediatric Institute, Üllői út 86, 1089 Budapest, Hungary

**Keywords:** Diseases, Endocrine system and metabolic diseases

## Abstract

Currently, there is no specific pharmaceutical agent for treating acute pancreatitis (AP). Somatostatin and its analogues have been used to prevent the autolysis of the pancreas in AP, however, their effectiveness has not been confirmed. This investigation aimed to examine the efficacy of ulinastatin, a protease inhibitor, combined with somatostatin analogues in the treatment of AP. We conducted a systematic database search in 4 databases to identify randomized controlled trials in which the efficacy of ulinastatin in combination with somatostatin analogue was compared to somatostatin analogue alone in patients with AP. Since the patient populations of analysed papers were slightly different, we used random effect models to pool odds ratios (OR) and mean differences (MD) and the corresponding 95% confidence intervals (CI). A total of 9 articles comprising 1037 patients were included in the meta-analysis. The combination therapy significantly reduced the complication rates for acute respiratory distress syndrome, acute kidney injury, and multiple organ dysfunction. Symptoms were relieved threefold with the combination therapy compared to somatostatin alone, and combination therapy significantly shortened the length of hospital stay. The decrease in mortality was not statistically significant.﻿

## Introduction

Acute pancreatitis (AP) is the sudden inflammation of the pancreas of various aetiologies, mainly alcohol and gallstones^1^. The incidence rate of AP ranges between 4.6 and 100 cases per 100,000 patients, however, its frequency has steadily increased in the past decade, especially in western countries^2,3^. The overall mortality rate is approximately 5%, but it is highly dependent on the disease severity^4^. Based on the Atlanta classification, AP can be categorized as mild, moderate, or severe depending on local and systemic complications^5^. Mild cases are primarily self-limiting and resolve within a week, but in severe cases the mortality can reach 20–40%^6^. Early identification and management of AP are crucial to achieve better patient outcomes. Treatment delay could lead to life-threatening complications even in cases of mild AP at onset. Currently, no specific pharmacological agents are targeting the pathophysiological mechanisms in AP. Only supportive therapies are available. International guidelines recommend early oral or enteral nutrition support, fluid therapy, and pain management^4,7–9^.

Somatostatin, and its more potent analogue octreotide, reduce pancreatic enzyme secretion, allowing the pancreas to rest and avoid further autodigestion^10^. However, clinical studies show no statistical difference in patient outcomes when comparing octreotide or somatostatin to placebo^11^. Even though international guidelines do not recommend somatostatin or octreotide, their use is common practice in the therapy of AP, especially in Asian countries^12^. Ulinastatin is a broad-spectrum serine protease inhibitor currently recommended by the Chinese authoritative guidelines and broadly used in many Asian countries for the treatment of acute pancreatitis^12^. However, a recent meta-analysis investigating mortality and adverse events of ulinastatin prescribed in AP did not find sufficient evidence to support its use^11^. Nevertheless, in theory, ulinastatin in combination with other agents might be useful in improving therapeutic efficiency.

The combination of ulinastatin with somatostatin or its analogue octreotide was tested in several clinical trials with promising results^13–15^, however the level of evidence is still low. Our systematic review and meta-analysis aimed to investigate the efficacy and safety of ulinastatin combined with somatostatin or octreotide in comparison with somatostatin derivatives alone in the management of acute pancreatitis.

## Methods

### Search strategy

For this systematic review and meta-analysis, we followed recommendations of the Cochrane collaboration^16^ and the Preferred Reporting Items for Systematic Reviews and Meta-Analyses (PRISMA) 2020 statement^17^. The review was registered in the International Prospective Register of Systematic Reviews (PROSPERO) database (registration number: CRD42021282614).

To answer the clinical question, we used the PICO framework. The population consisted of adult patients (> 18 years old) with acute pancreatitis; the intervention group included patients who received the combination treatment (ulinastatin therapy with somatostatin or octreotide) besides other supportive measures; the control or comparator group included cases treated with somatostatin or octreotide monotherapy besides other supportive measures. The primary outcomes were mortality, complications—Acute Respiratory Distress Syndrome (ARDS), shock, Acute Kidney Injury (AKI), Multiple Organ Dysfunction Syndrome (MODS), and length of hospital stay. As secondary outcomes, we evaluated symptom reduction rate, changes in laboratory parameters, and adverse events of the intervention.

The search was performed on 15 November 2021 in four databases (PubMed, Embase, Web of Science, and the Cochrane Central Register of Controlled Trials) to identify the randomized clinical trials meeting the previously mentioned eligibility criteria. The search key was: pancreatitis AND ulinastatin AND (octreotide or octreotid* or somatostatin) AND random*, and we did not use restrictions or filtering options. We used Google Translate® for translation of articles in languages other than English or German. Plot Digitizer (2015) was used to transform graphical values into numerical form. We additionally searched the reference list of the included studies.

### Selection and data collection process

The search results were exported to the EndNote X9 citation manager (Clarivate Analytics, Philadelphia, PA, USA). After the automatic and manual duplicate removal (ILH), the title and abstract, and full-text selection processes were done by two independent authors according to the inclusion criteria (ILH and DK). A third author (DC) made the final decision in case of disagreements. Cohen’s kappa coefficient was calculated at each selection step to evaluate the level of agreement between the authors. Two independent investigators (ILH and DK) manually extracted the data from the eligible articles and cross-checked each other’s data sets to ensure precision. The following data were extracted: study characteristics (first author, year of publication, country, number of centres, setting), population description (sample size, percentage of female participants, age, AP severity), therapy details (drug type, dose, regimen, duration), and outcomes as reported in each article. Microsoft Excel (Microsoft, Office 365, Redmond, WA, USA) was used for data collection.

### Statistics

We used the methods recommended by the working group of the Cochrane Collaboration^23^ for data synthesis. Only outcomes reported in at least three studies were considered for including in the meta-analysis. The pooled results were reported as ORs (odds ratios) for binary outcomes calculated with the Mantel–Haenszel method, and as mean differences (MDs) for continuous outcomes and the corresponding 95% confidence intervals (CI). In case of binary outcomes, ORs were used for the effect measure, while for continuous outcomes MDs with corresponding standard deviations (SDs) were used. In the latter case when only before-and-after treatment group means and SDs were reported, we used the difference in means, and the sum of within-group before-and-after SDs as a conservative estimate for SDs of the differences. For binary outcomes, raw data from the selected studies were pooled with the Mantel–Haenszel method, while for continuous outcomes mean differences were calculated. Random models were used for pooling in case of both outcome types. Subgroup comparisons were carried out following the description in Harrer et al.^18^. To estimate τ^2^ we used the Paule-Mandel method and the Q profile method for calculating the confidence interval of τ^2^ ^18,19^. A funnel plot of the logarithm of effect size and comparison with the standard error for each trial was used to evaluate publication bias. Statistical heterogeneity across trials was assessed by means of the Cochrane Q test and the I^2^ statistic values^20^. I^2^ values of 25, 50, and 75% were identified as low, moderate, and high estimates, respectively. Outlier and influence analyses were carried out following the recommendations of Harrer et al. and Viechtbauer and Cheung^18,21^. Forest plots were used to graphically summarize results^22,23^. Where applicable, we reported the prediction intervals (i.e., the expected range of effects of future studies) of results following the recommendations of IntHout et al.^23^.

All analyses were carried out in R version 4.1.3 (R Core Team, Vienna, Austria) using the meta^24^ and dmetar^18^ packages.

### Risk of bias assessment

The risk of bias assessment was performed by two independent authors (ILH and DK) using the revised Cochrane risk-of-bias tool (RoB2)^25^, while disagreements were solved by consensus. The domains evaluate the bias arising from the randomization process, deviations from the intended intervention, missing data, the measurement of the outcome, and the selection of the reported results. The final conclusion of the risk assessment could be characterized as ‘low’, ‘some concerns’, or ‘high’.

### GRADE

We used the Grading of Recommendations, Assessment, Development and Evaluations (GRADE) framework to evaluate the level of evidence for our findings^26^. Each outcome was rated for risk of bias, inconsistency, indirectness, imprecision, publication bias, and the presence of a large effect, dose-dependent response, and plausible confounders as ‘not serious’, ‘serious’, or ‘very serious’. The final certainty of the evidence was categorized as ‘very low’, ‘low’, ‘moderate’, or ‘high’.

### Ethical approval

No ethical approval was required for this systematic review with meta-analysis, as all data were already published in peer-reviewed journals. No patients were involved in the design, conduct or interpretation of our study.

## Results

### Description of included studies

**Figure 1:**
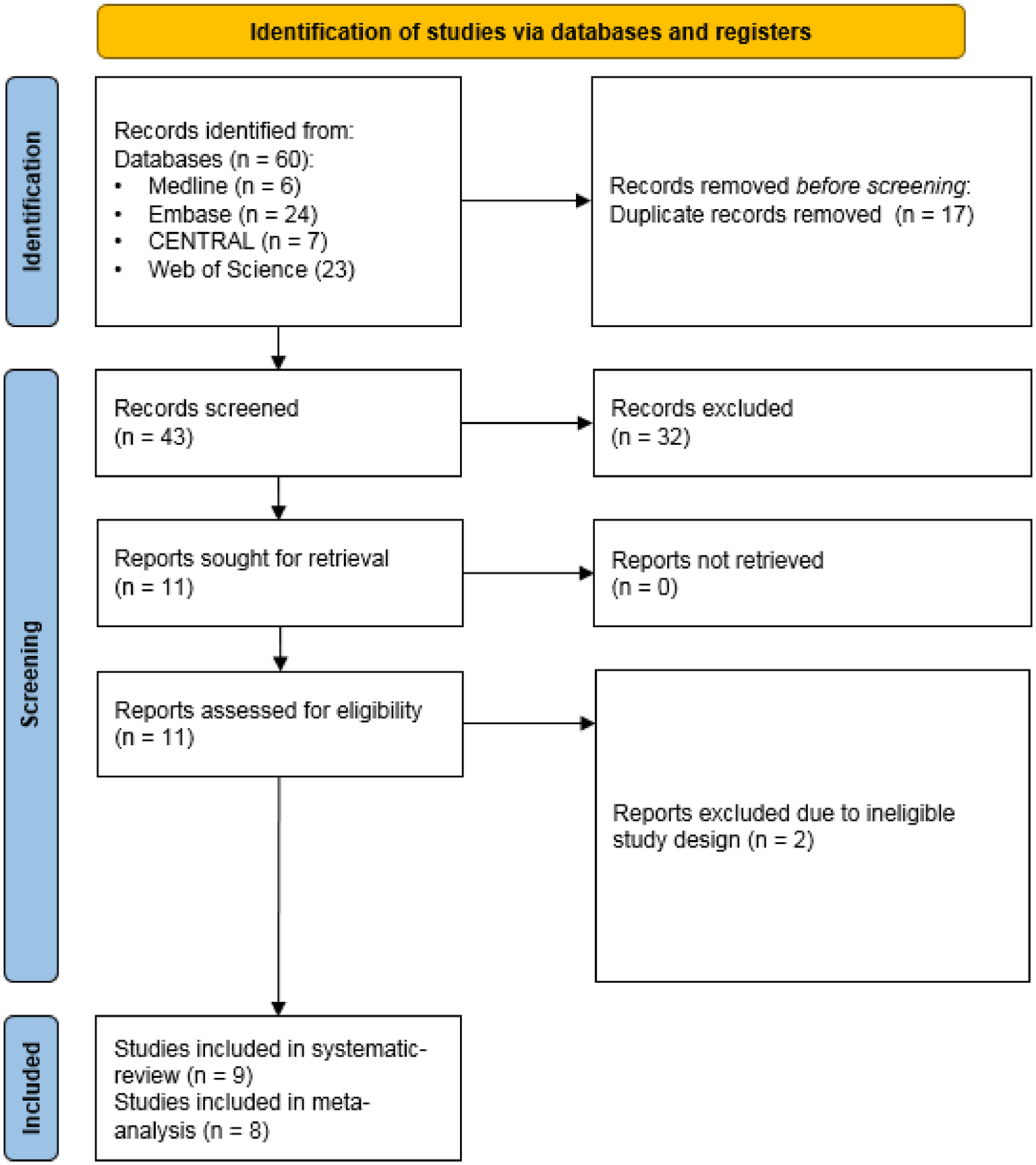
PRISMA flowchart.

The database search identified 60 records. After duplicate removal, and title and abstract selection (Cohen’s Kappa 0.93), we identified 9 eligible articles during the full-text article analysis (Cohen’s Kappa 1.00). All included reports were available as peer reviewed journal articles. The search results and the selection process are summarized in Fig. 1.

Overall, 9 studies were included in our systematic review. There were no overlapping populations in the meta-analyses. All studies were single centre. Treatment arm allocation ratios were 1:1 in each study. The baseline characteristics of eligible studies are summarized in Table 1. The posology for each therapeutic regimen is detailed in Table 2.

**Table 1:** Baseline characteristics of the included trials.

Study	Country	Population	Sample size (% female)	Intervention group	Sample size [intervention group] (% female)	Mean age (years) ± SD [intervention group]	Control group	Sample size [control group] (% female)	Mean age (years) ± SD [control group]	Outcomes
Wang et al. (2013)^27^	China	Severe acute pancreatitis	123 (49.6)	Ulinastatin + octreotide	62 (50.0)	41.8 ± 13.9	Somatostatin	61 (49.2)	42.6 ± 12.6	Mortality; MODS
Tu et al. (2014)^28^	China	Acute pancreatitis	110 (47.3)	Ulinastatin + octreotide	55 (45.5)	37.3 ± 6.1	Octreotide	55 (49.1)	38.7 ± 5.8	LOH; SR; APR
Guo et al. (2015)^13^	China	Severe acute pancreatitis	120 (46.7)	Ulinastatin + octreotide	60 (48.3)	46.6 ± 4.1	Octreotide	60 (45.0)	46.3 ± 4.3	Mortality; LOH; MODS; ARDS AKI; shock; SR; APR
Wang et al. (2016)^14^	China	Severe acute pancreatitis	246 (48.8)	Ulinastatin + octreotide	124 (49.2)	40.8 ± 11.6	Somatostatin	122 (48.4)	41.9 ± 12.8	Mortality; LOH; MODS; SR; APR
Wang et al. (2017)^30^	China	Moderateliy severe and severe acute pancreatitis	42 (40.5)	Ulinastatin + octreotide	21 (42.9)	47.3 ± 11.1	Somatostatin	21 (38.1)	48.6 ± 10.0	ARDS; AKI; shock; APR
Yang et al. (2017)^32^	China	Severe acute pancreatitis	88 (39.8)	Ulinastatin + octreotide	44 (40.9)	42.1 ± 9.8	Octreotide	44 (38.6)	43.2 ± 9.2	N/A
Yang et al. (2018)^15^	China	Severe acute pancreatitis	94 (37.2)	Ulinastatin + octreotide	46 (41.3)	46.2 ± 10.6	Octreotide	48 (33.3)	47.7 ± 11.8	Mortality; LOH; ARDS; AKI; shock; SR; APR
Meng et al. (2019)^31^	China	Acute pancreatitis	108 (45.4)	Ulinastatin + octreotide	54 (N/A)	N/A	Octreotide	54 (N/A)	N/A	SR
Xu et al. (2019)^29^	China	Severe acute pancreatitis	106 (49.1)	Ulinastatin + octreotide	53 (50.9)	57.0 ± 6.9	Somatostatin	53 (47.2)	57.5 ± 7.4	LOH; SR

SD, standard deviation; N/A, not reported; MODS, multiple organ dysfunction syndrome; LOH, length of hospital stay; SR, symptom reduction; APR, abdominal pain relief; ARDS, acute respiratory distress syndrome; AKI, acute kidney injury.

**Table 2:** Summary of the applied therapies as reported in each eligible article.

Study	Intervention group	Dose	Regime	Duration (days)	Control group	Dose	Regime	Duration (days)
Wang et al. (2013)^27^	Ulinastatin + somatostatin	100000 U	q12h	10	Somatostatin	250 mcg/h	Continuous	10
Tu et al. (2014)^28^	Ulinastatin + octreotide	200000 U	qd	14	Octreotide	0.5 g/(kg x h)	N/A	14
Guo et al. (2015)^13^	Ulinastatin + octreotide	(1) 100000 U (2) 50000 U	(1) q12h (2) q12h	(1) for 3 (2) then 7–14	Octreotide	0.1 mg	q8h	7–14
Wang et al. (2016)^14^	Ulinastatin + somatostatin	100000 U	q12h	10	Somatostatin	3 mg	Continuous	10
Wang et al. (2017)^30^	Ulinastatin + somatostatin	100000U	(1) q12h (2) q24h	(1) for 3 (2) then 7	Somatostatin	6 mg	Continuous	10
Yang et al. (2017)^32^	Ulinastatin + octreotide	100000 U	q12h	10	Octreotide	0.1 mg	q6h	7
Yang et al. (2018)^15^	Ulinastatin + octreotide	200000 U	qd	14	Octreotide	0.1 mg bolus + 25 mcg/h	Continuous	14
Meng et al. (2019)^31^	Ulinastatin + octreotide	100000U	q12h	7	Octreotide	0.6 mg	Continuous	7
Xu et al. (2019)^29^	Ulinastatin + somatostatin	100000 U	q24h	7	Somatostatin	6 mg	Continuous	7

U, unit; q, every; h, hour; d, day; mcg, microgram; mg, milligram, N/A, not reported.

The following outcomes were eligible for meta-analysis: mortality in 4 trials^13–15,27^; length of hospital stay in 5 trials^13–15,28,29^; multiple organ dysfunction syndrome in 3 trials^13,14,27^; acute respiratory distress syndrome in 3 trials^13,15,30^; acute kidney injury in 3 trials^13,15,30^; shock in 3 trials^13,15,30^; symptom reduction in 6 trials^13–15,28,29,31^; and abdominal pain relief in 5 trials^13–15,28,30^; CRP change in 6 trials^13,15,28–30,32^. We reported the results of Yang et al.^32^ in the systematic review since they only assessed laboratory parameters, which were insufficient for further statistical analysis.

### Primary outcomes

#### Complication rates

**Figure 2:**
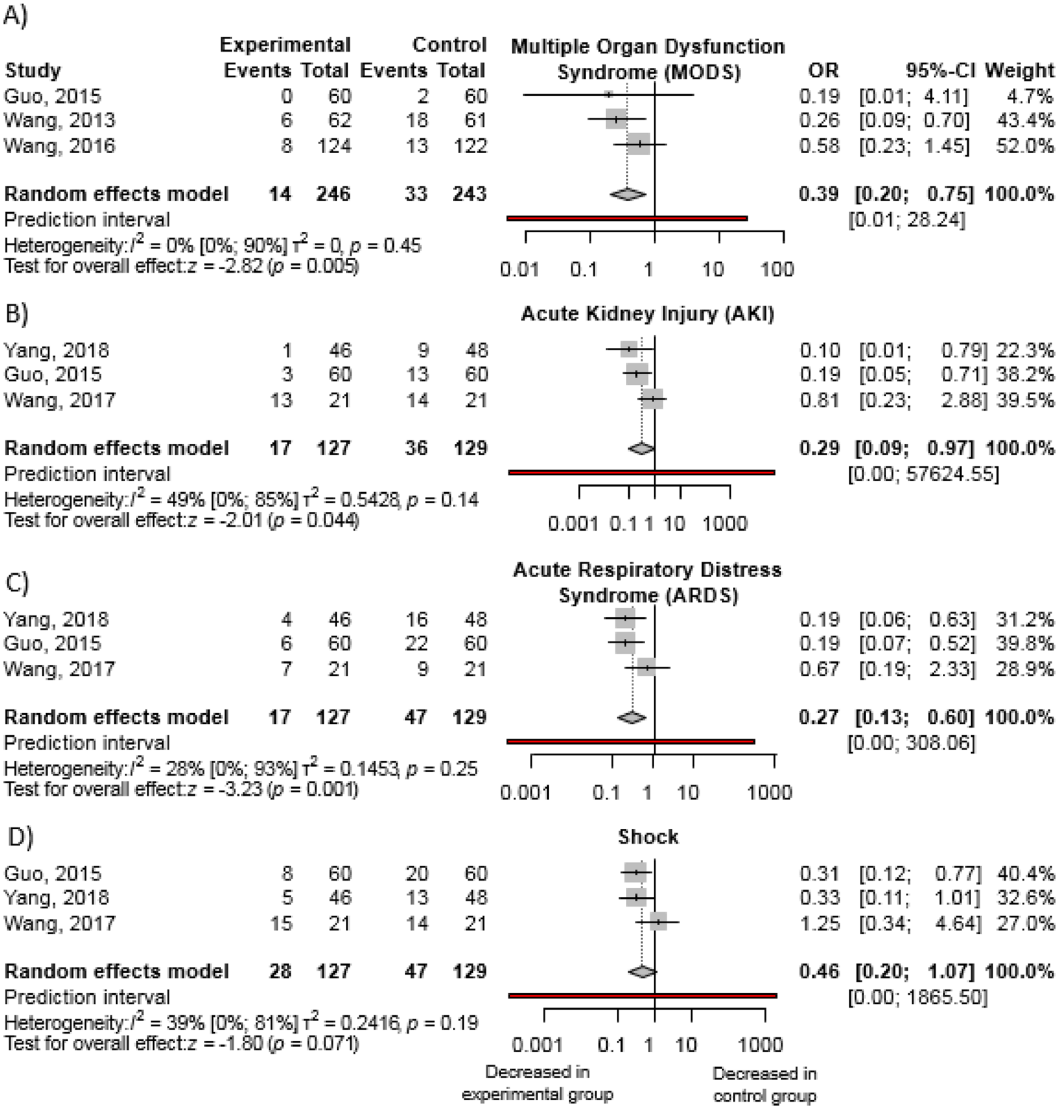
Ulinastatin in combination with somatostatin analogue decreases rates of: (**a**) MODS, (**b**) AKI, and (**c**) ARDS, but not of (**d**) shock, compared to somatostatin analogue monotherapy when administered besides standard of care in acute pancreatitis. (OR, odds ratio; CI, confidence interval).

Our pooled results revealed decreased complication rates in the intervention group (Fig. 2). With the combination therapy, rates of ARDS [OR 0.27; 95% CI 0.13–0.60; I^2^ = 28%] and AKI [OR 0.29; 95% CI 0.09.-0.97; I^2^ = 49%] were reduced by approximately 70%, while MODS could be prevented in around 60% of cases [OR 0.39; 95% CI 0.20–0.75; I^2^ = 0%]. Reduction of shock incidence was not statistically significant [OR 0.46; 95% CI 0.20–1.07; I^2^ = 39%]. The associated heterogeneity for the results was not important or moderate, however, due to the low number of trials, interpretation has to be treated with caution.

#### Mortality

**Figure 3:**
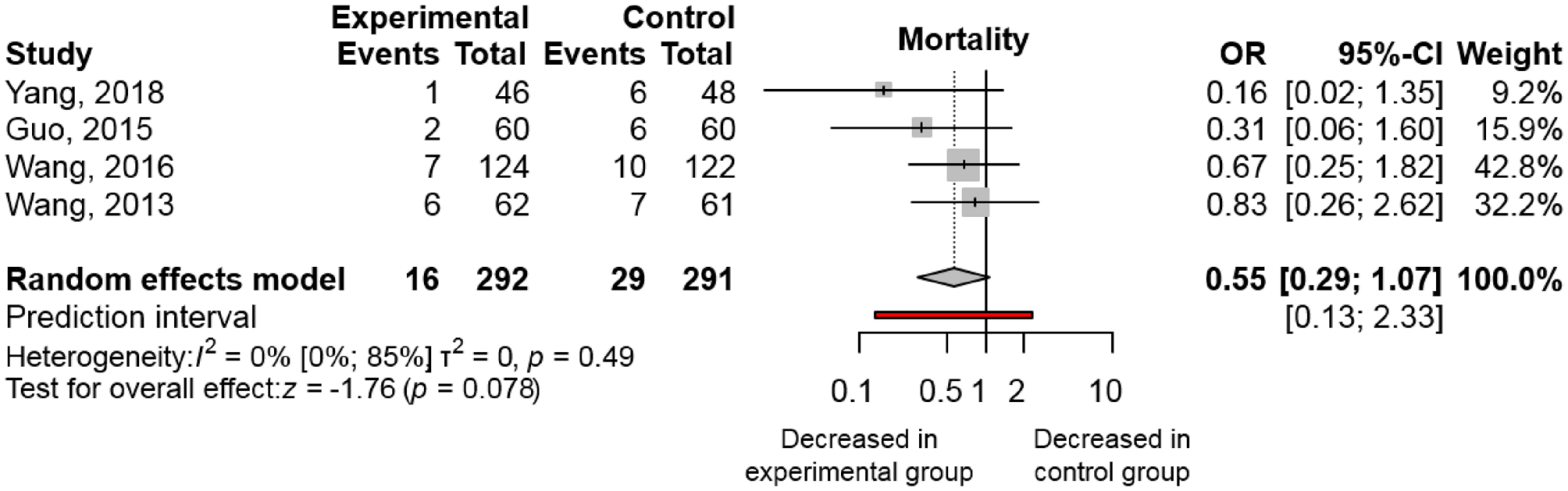
Ulinastatin in combination with somatostatin analogue is associated with decreasing trends in mortality when compared to somatostatin analogue monotherapy. (OR, odds ratio; CI, confidence interval).

Analysis of pooled data from 4 trials^13–15,27^, including 583 patients, shows a tendency for a decreased mortality rate with the combination therapy [OR 0.55; 95% CI 0.29–1.07; I^2^ = 0%]; however, the result was not statistically significant (Fig. 3). These studies yielded homogenous results. All studies reported on in-hospital mortality.

#### Length of hospital stay

**Figure 4:**
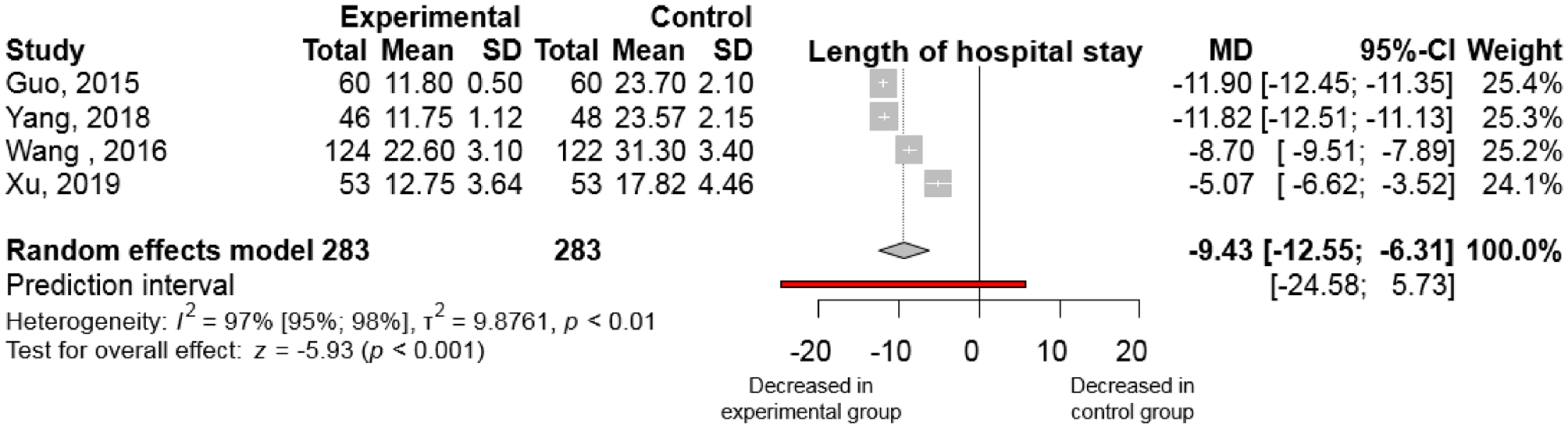
Ulinastatin combination with somatostatin analogue administered besides standard of care decreases the length of hospital stay in severe acute pancreatitis cases by comparison with somatostatin alone. (MD, mean difference; CI, confidence interval).

Four studies^13–15,28,29^ reported the length of hospital stay, measured in days. In the intervention group, admission duration was shortened by 9.43 days [95% CI (-12.55)-(-6.31); I^2^ = 97%] by comparison with the control group (Fig. 4). The results showed substantial heterogeneity. The effect was similar for severe AP cases [MD (− 8.10); 95% CI (− 11.64) to (− 4.56); I^2^ = 99%; Fig. 3S].

### Secondary outcomes

The definition of treatment effectiveness varied across the included studies. The common elements of these definitions were (a) reduction of pancreatitis symptoms; abdominal pain, nausea, vomiting, (b) normalization of laboratory parameters evaluated at certain time intervals after treatment initiation. The time of evaluation varied among the included studies. The therapy was considered ineffective if the patients’ symptoms or laboratory parameters were not improved. A summary of effectiveness definitions in each study is available in Table 1S.

#### Symptom reduction

Six trials^13–15,28,29,31^, including 651 patients, reported symptom reduction. Among the assessed symptoms were gastrointestinal manifestations and abdominal pain, as well as laboratory parameters. They were evaluated at 7–17 days from treatment start. Pooled analysis shows 3.51 times higher odds of symptoms reduction in the combined therapy group than in the monotherapy group [OR 3.51; 95% CI 2.30–5.37; I^2^ = 0%; Fig. 4S]. This effect is similar in the subgroup analysis of the severe cases [OR 3.32; 95% CI 2.07–5.33; I^2^ = 0%; Fig. 4S].

#### Abdominal pain relief

**Figure 5:**
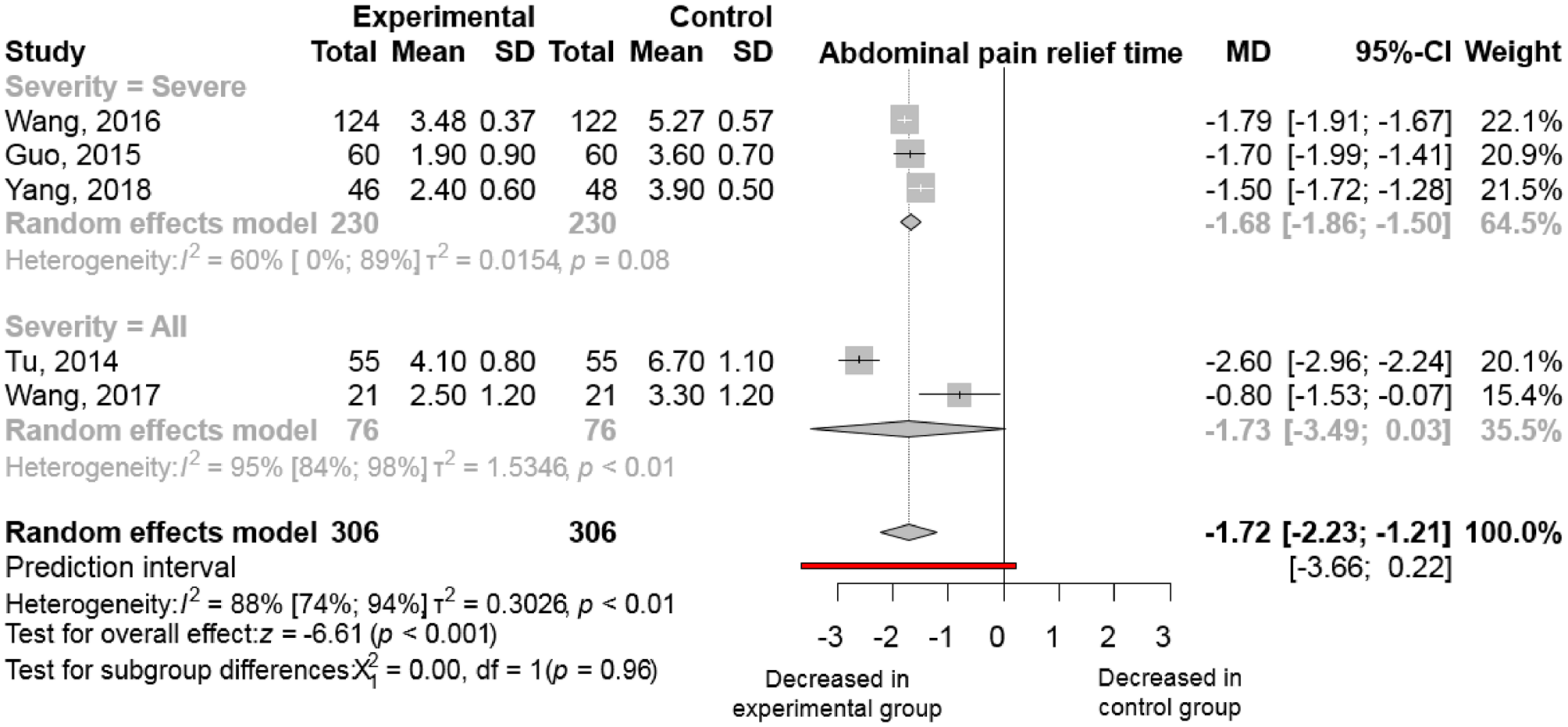
Ulinastatin in combination with somatostatin analogue decreases time to abdominal pain relief. (MD, mean difference; CI, confidence interval).

Duration until abdominal pain relief was specifically reported in 5 trials^13–15,28,30^, including 612 patients. It was measured as the number of days patients reported abdominal pain. Ulinastatin combined with somatostatin analogue led to significantly faster pain relief than somatostatin derivates monotherapy. The mean difference is − 1.72 days [95% CI (− 2.23) to (− 1.21); I^2^ = 88%, Fig. 5]. The results were similar in the severe form of acute pancreatitis [MD − 1.68; 95% CI (− 1.86) to (− 1.50); I^2^ = 60%; (Fig. 5)].

### Additional outcomes

Some of the studies reported on variations from baseline in several laboratory parameters, of which we were able to meta-analyse the results for C-reactive protein (CRP). There was a significant difference between the two groups regarding the reduction in CRP values from baseline to the end of treatment [MD 13.73 mmol/L, 95% CI 4.44–23.02; I^2^ = 73%], favouring the intervention group (Fig. 5S). Although we could not include the results for the other laboratory parameters (amylase, white blood cell count, TNFα, interleukins (Il-6, -8, -10), diamine oxidase) in our meta-analysis, the identified trends favoured the combination therapy. These results are summarized in the supplementary material (Tables 2S–10S).

### Risk of bias assessment and quality of evidence

The overall risk of bias was moderate, mainly due to inaccurate reporting of blinding, imprecise measure reporting, and lack of available study protocols. The quality of evidence was low to moderate because of the small sample sizes and the overall moderate bias. The detailed results of the risk of bias assessment and the summary of findings table for GRADE are presented in the supplementary material (Figs. 1S, 2S, and Table 11S, respectively).

Publication bias could not be assessed due to an insufficient number of studies.

## Discussion

### Principal findings

Our meta-analysis assessed the clinical advantage of the combination therapy of ulinastatin with somatostatin analogues compared to somatostatin alone besides standard of care in acute pancreatitis. The ulinastatin combined with somatostatin or octreotide therapy significantly reduced the majority of systemic complications rates, the systemic inflammation as reflected by the significant improvement in the laboratory parameters, the length of hospital stay and the time to abdominal pain relief compared to somatostatin alone. Data about mortality and shock rates are limited.

Our results indicate that the intervention determines a threefold symptom reduction compared with monotherapy, which is consistent in severe acute pancreatitis. The better response rate might be a contributing factor to a faster recovery and to avoid complications. It could alleviate abdominal pain almost 2 days earlier than monotherapy. Abdominal pain is the leading symptom of AP; adequate management has a great impact on patients’ perspectives^33^. Moreover, the combination therapy could significantly reduce CRP, thus decreasing the inflammation. With fewer days of hospital stay and lower complication rates, it is a clinically effective therapy. Additional health care expenses could be spared in both short- and long-term.

Mortality showed a decreasing trend in the experimental group, but the results were not statistically significant. If we expect a reduction in mortality of 10% (from 12 to 2%) within the intervention group^15,34^ an optimal study sample size would be approximately 99 patients in each study arm (80% power, one-sided alpha level of 5% with continuity correction). None of the studies reached this threshold, so our results must be considered cautiously since we cannot strongly confirm the impact of the combination therapy on mortality.

The development of acute pancreatitis is initiated by excess Ca^2+^ signal generation, which leads to decreased mitochondrial ATP generation in the acinar cells, and promotes the activation of trypsin, resulting in necrosis^35^. An in vitro study by Kanayama^36^ suggests that ulinastatin might inhibit Ca^2+^ influx or mobilization, however, this effect has not been studied further. If given early, ulinastatin, a trypsin inhibitor, may suppress the trypsin autoactivation sequence. Furthermore, it also inhibits chymotrypsin, thrombin, kallikrein, neutrophil elastase, and cathepsin, thereby regulating systemic inflammation by reducing release of pro-inflammatory cytokines^37^. Moreover, ulinastatin inhibits necrosis by preventing mitochondrial damage, decreases endothelial dysfunction, normalizes coagulation disturbances, improves perfusion, and thereby restores organ functions^37–40^. This complex mechanism of action might complement those of somatostatin analogues explaining the increased efficacy of the combination treatment in acute pancreatitis. In hereditary pancreatitis, activation of trypsinogen has a pathogenic role in the development of chronic pancreatitis after an acute AP episod﻿e^41,42^. Further investigations are needed for the precise mechanism of action.

Several meta-analyses revealed positive effects of ulinastatin in many severe clinical scenarios: it can prevent postoperative bleeding in patients undergoing cardiac surgery^43^, it protects against ischemia–reperfusion injuries in hepatectomy^44^, in ARDS of various etiologies it decreases the mortality rates^45^, after cardiopulmonary bypass it reduces pulmonary injury and improves pulmonary function^46^, and decreases the duration of mechanical ventilation^47^. The clinical effects of ulinastatin observed in patients suffering from diseases that associate high risk of major complications come to support its potential in the management of acute pancreatitis.

### Strength and limitations

To the best of our knowledge, this is the first meta-analysis on this topic. The strength of this review is its rigorous methodology. We strictly followed the Cochrane and PRISMA recommendations and ensured the study’s transparency through the prior publication of the review protocol on PROSPERO.

However, we identified several limitations. The conclusions are based on a limited number of trials performed only in China. Due to the small sample sizes, interpretation must be made carefully. There was no mention of sample size calculation in the trials. These factors resulted in high heterogeneity in some cases. Furthermore, variability in the population, and the differences in the applied treatment durations, doses, and follow-up times were also major contributing factors to the high heterogeneity. The included trials are of low to moderate quality, with the risks of bias resulting from a lack of proper reporting of blinding participants and investigators. Furthermore, there were no available study protocols to assess the intended and reported outcomes.

### Implications for research and clinical practice

Somatostatin analogue monotherapy is not sufficiently effective in the therapy of AP. Although the results presented here suggest an improvement of efficacy when combined with ulinastatin, this combination should be further studied e.g., to overcome the limitation that all the available data are available from trials performed in China. Because of the differences in the applied treatments, outcome measures, and follow-up time, further multicentre, double-blind, randomized controlled clinical trials with greater sample sizes and well-defined outcomes are needed to assess the combination therapy's effect in acute pancreatitis. Moreover, data on the safety of the combination therapy in AP are missing. Because of the shorter hospital stay and decreased complications risk, cost-effectiveness and health technology assessment should be considered. The clinical efficacy and safety of further combination therapies should be assessed systematically.

This meta-analysis provides new insight into a possible drug therapy treatment for acute pancreatitis. This is especially important in severe cases, as there are limited treatment options and the mortality is high.

## Conclusion

Ulinastatin combined with somatostatin analogue significantly decreased complication rates (ARDS, AKI, MODS) in AP in comparison with somatostatin analogue monotherapy. Moreover, combination therapy is associated with earlier symptoms relief and shorter hospital stay. Further RCTs of larger sample sizes would accurately evaluate the effect of this combination therapy.

## Supplementary Information

Supplementary Information 1.

Supplementary Information 2.

## Data Availability

The datasets used and/or analysed during the current study available from the corresponding author on reasonable request.
