# Supplementary material for: The combination of ulinastatin and somatostatin reduces complication rates in acute pancreatitis: a systematic review and meta-analysis of randomized controlled trials
Source: Sci Rep. 2022 Oct 26;12:17979. doi: 10.1038/s41598-022-22341-7 (PMC9606296; doi:10.1038/s41598-022-22341-7)
Supplement: Supplementary file 2 — Supplementary Information 2. [file 41598_2022_22341_MOESM2_ESM.docx]

Supplementary material

**The combination of ulinastatin and somatostatin reduces complication rates in acute pancreatitis: a systematic review and meta-analysis of randomized controlled trials**

István László Horváth^1,2^, Stefania Bunduc^1,4,6,7^, Péter Fehérvári^1,8^, Szilárd Váncsa^1,4^, Rita Nagy^1,3,11^, Gantsetseg Garmaa^1,9^, Dénes Kleiner^1,2^, Péter Hegyi^1,3,4,5^, Bálint Erőss^1,3,4,5^, Dezső Csupor^1,3,10,*^

**Affiliations**

^1^ Centre for Translational Medicine, Semmelweis University, 1085 Budapest, Üllői út 26, Hungary

^2^ University Pharmacy Department of Pharmacy Administration, 1092 Budapest, Hőgyes Endre utca 7-9.

^3^ Institute for Translational Medicine, Medical School, University of Pécs, 7624 Pécs, Szigeti út 12, Hungary

^4^ Division of Pancreatic Diseases, Heart and Vascular Center, Semmelweis University, 1085 Budapest, Baross út 22-24, Hungary

^5^ János Szentágothai Research Center, University of Pécs, 7624 Pécs, Szigeti út 12, Hungary

^6^ Carol Davila University of Medicine and Pharmacy, 020021 Bucharest, Dionisie Lupu street 37, Romania

^7^ Fundeni Clinical Institute, 022328 Bucharest, Fundeni street 258, Romania

^8^ University of Veterinary Medicine, Budapest Department of Biomathematics and Informatics, 1078 Budapest, István utca 2

^9^ Institute of Translational Medicine, Semmelweis University, 1089 Budapest, Nagyvárad tér 4., Hungary

^10^ Institute of Clinical Pharmacy, University of Szeged, 6725 Szeged, Szikra utca 8, Hungary

^11^ Heim Pál National Pediatric Institute, 1089 Budapest, Üllői út 86, Hungary

*corresponding author


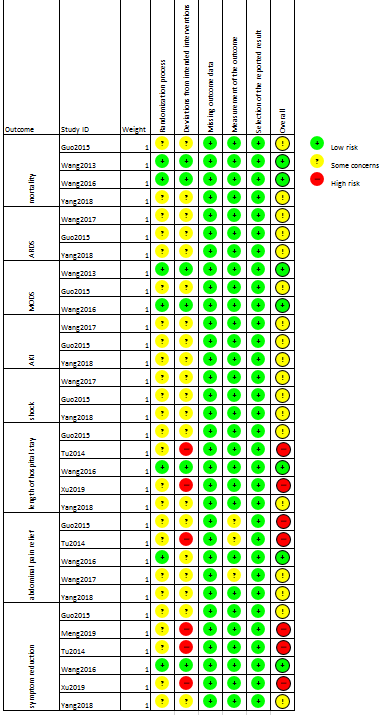


Figure 1S. Risk of bias assessment of each outcome using the revised Cochrane risk-of-bias tool (RoB2)

Figure 2S. Summary for the risk of bias assessment using the revised Cochrane risk-of-bias tool (RoB2)

| Tu et al (2014) | (1) Recovery: After treatment, the clinical symptoms of the patients have returned to normal, and the complications have completely disappeared. Pancreatic colour, Doppler ultrasound examination results also returned to normal. At the same time, urine amylase also showed normal;  (2) Significantly effective: the above-mentioned clinical symptoms improved after 3 days of treatment, but did not return to normal;  (3) Effective: the above-mentioned symptoms after 1 week of treatment The clinical symptoms have been improved, but have not returned to normal;  (4) Ineffective: After 1 week of treatment, the above clinical symptoms have not been improved or even worsened |
| --- | --- |
| Guo et al (2015) | (1) Cure means that the patient’s abdominal pain and bloating symptoms disappeared within 3 days after treatment, no symptoms of nausea and vomiting, upper abdominal tenderness disappeared, and laboratory indicators returned to normal;  (2) markedly effective means that the patient’s abdominal pain and abdominal distension disappeared within 7 days of treatment, no nausea and vomiting symptoms, upper abdominal tenderness disappeared, and laboratory indicators returned to normal;  (3) effective means that the patient’s abdominal pain and abdominal distension disappeared within 10 days after treatment, no nausea and vomiting symptoms, upper abdominal tenderness disappeared, and laboratory indicators returned to normal;  (4) Ineffective means that the patient's indicators have not improved or deteriorated more than 10 days after treatment. |
| Wang et al (2016) | Cure rate |
| Meng et al (2019) | (1) Excellent was labelled if the symptoms and signs such as nausea, vomiting, abdominal distension and abdominal pain disappeared, and the urinary amylase has been reduced to the normal range or the reduction is more than 3/4. (2) Effective was labelled when the relevant clinical symptoms and signs markedly improved and the urinary amylase reduced by more than 1/2.  (3) It was non-effective when there was no remarkable changes in the relevant clinical symptoms and signs, or even worsening; urinary amylase did not markedly reduced or complications developed |
| Xu et al (2019) | (1) Significant improvement: After treatment, SAP related symptoms and signs disappeared. Serum amylase returned to normal.  (2) Improvement: After treatment, SAP related symptoms and signs improved. Serum amylase improved but did not return to normal.  (3) Ineffectiveness: After treatment, SAP related symptoms and signs were not improved or aggravated. Serum amylase was not significantly reduced |

Table 1S. Effectiveness definition as provided in each eligible article


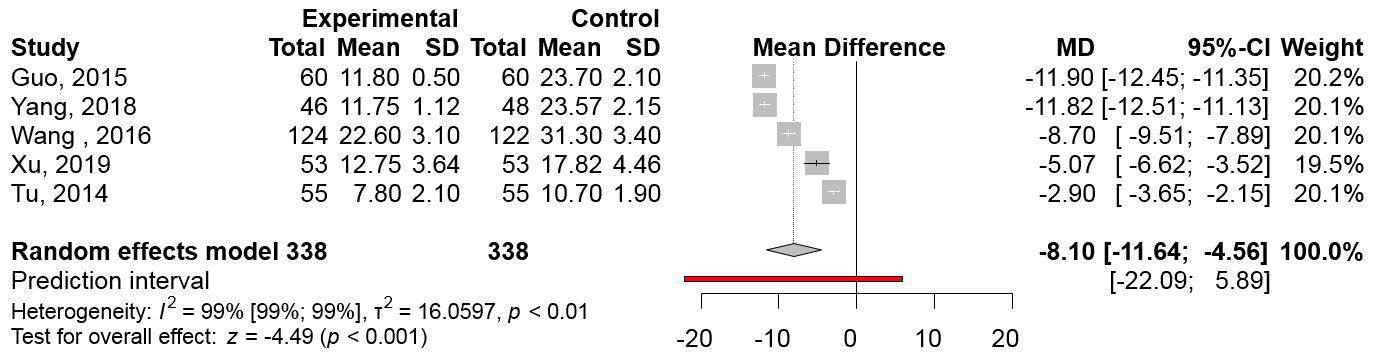


Figure 3S. Ulinastatin combination with somatostatin analogue decreases the length of hospital stay in all severity forms of acute pancreatitis


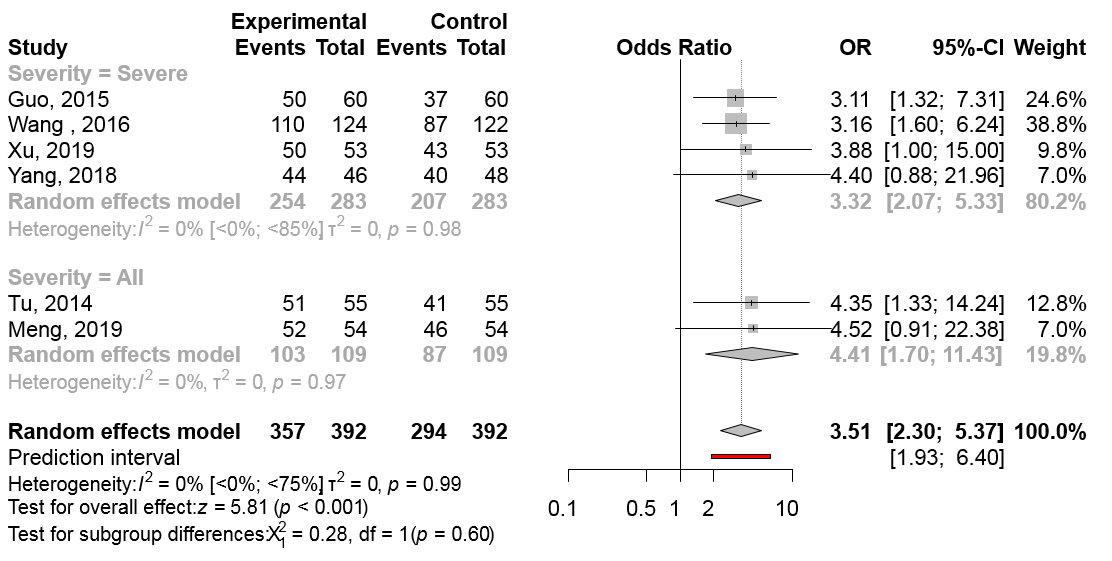


Figure 4S. Ulinastatin combination with somatostatin analogue increases the odds of symptom reduction


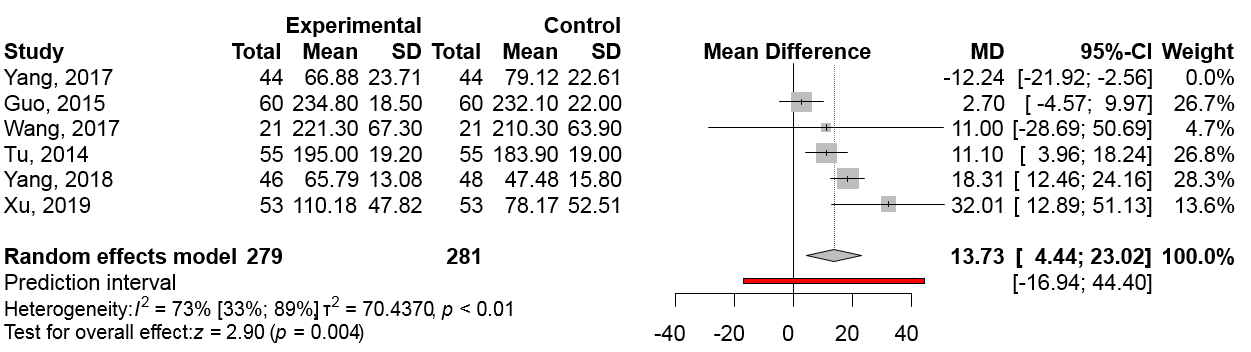


Figure 5S. Ulinastatin combination with somatostatin analogue has greater effect on CRP value than monotherapy


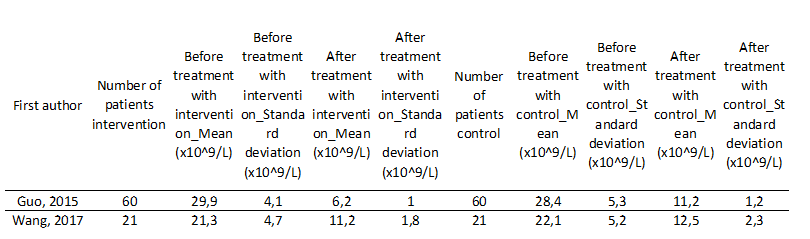


Table 2S. Serum level of White Blood Cells before-after treatments


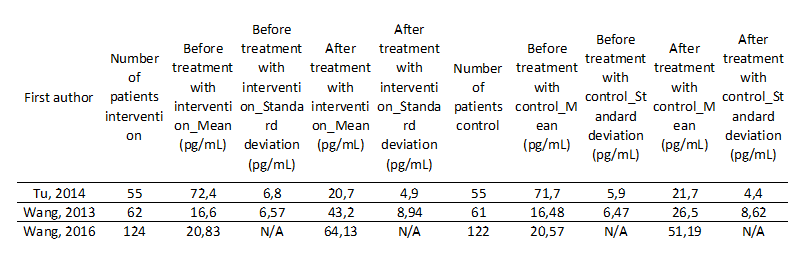


Table 3S. Serum level of Il-10 before-after treatments


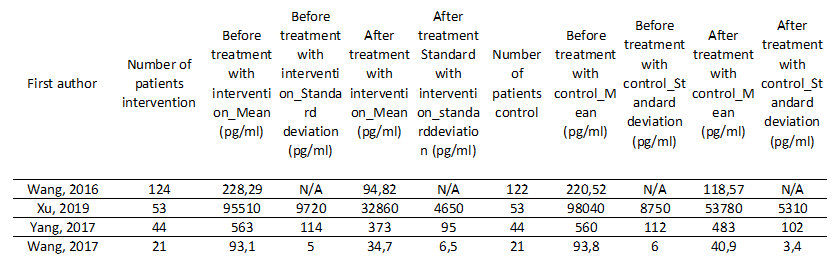


Table 4S. Serum level of Il-8 before-after treatments


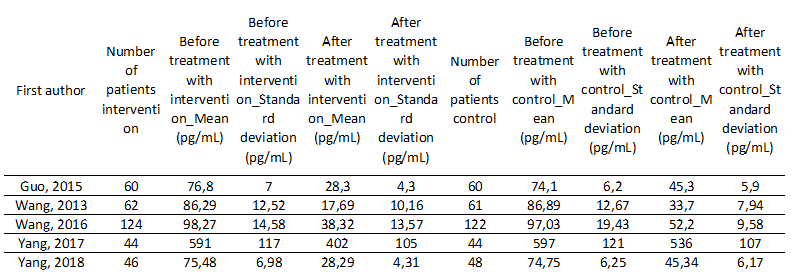


Table 5S. Serum level of Il-6 before-after treatments


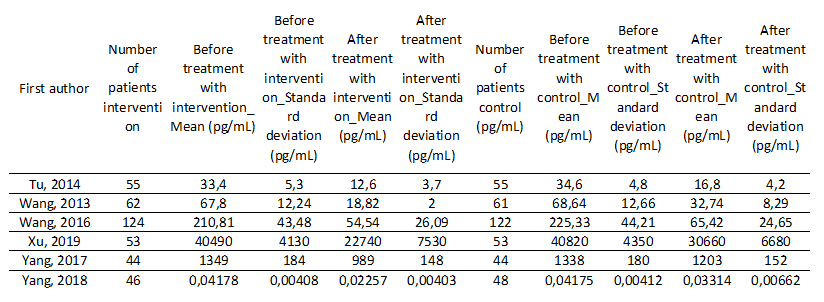


Table 6S. Serum level of Tumor necrosis factor alfa before-after treatments


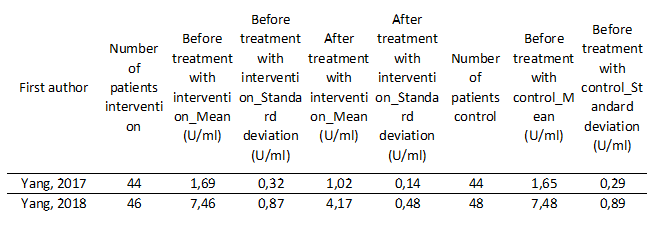


Table 7S. Serum level of Diamino-oxidase before-after treatments


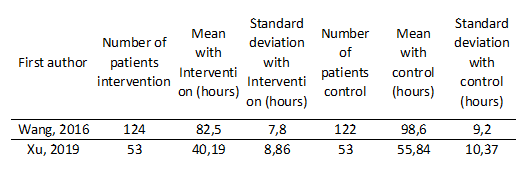


Table 8S Normalization time of blood amylase


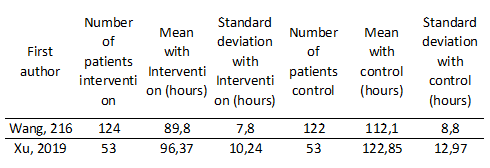


Table 9S. Normalization time of respiration


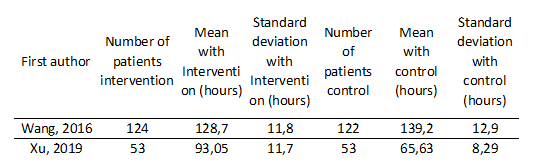


Table 10S. Normalization time of heart rate

| **Certainty assessment** | | | | | | | **№ of patients** | | **Effect** | | **Certainty** | **Importance** |
| --- | --- | --- | --- | --- | --- | --- | --- | --- | --- | --- | --- | --- |
| **№ of studies** | **Study design** | **Risk of bias** | **Inconsistency** | **Indirectness** | **Imprecision** | **Other considerations** | **ulinastatin combination with somatostatin analogue** | **somatostatin analogue** | **Relative (95% CI)** | **Absolute (95% CI)** |  |  |
| **mortality** | | | | | | | | | | | | |
| 4 | randomised trials | not serious | not serious | not serious | serious^a^ | none | 16/292 (5.5%) | 29/291 (10.0%) | **OR 0.55** (0.21 to 1.44) | **4 fewer per 100** (from 8 fewer to 4 more) | ⨁⨁⨁◯ Moderate | CRITICAL |
| **Length of hospital stay** | | | | | | | | | | | | |
| 5 | randomised trials | not serious | not serious | not serious | serious^a^ | none | 338 | 338 | - | MD **8.1 SD lower** (11.64 lower to 4.56 lower) | ⨁⨁⨁◯ Moderate | CRITICAL |
| **Lenght of hospital stay_severe** | | | | | | | | | | | | |
| 4 | randomised trials | not serious | not serious | not serious | serious^a^ | none | 283 | 283 | - | MD **9.43 lower** (12.55 lower to 6.31 lower) | ⨁⨁⨁◯ Moderate | CRITICAL |
| **Effectiveness** | | | | | | | | | | | | |
| 6 | randomised trials | serious | not serious | not serious | serious^a^ | none | 357/392 (91.1%) | 294/392 (75.0%) | **OR 3.51** (2.95 to 4.18) | **16 more per 100** (from 15 more to 18 more) | ⨁⨁◯◯ Low | IMPORTANT |
| **Effectivemess_severe** | | | | | | | | | | | | |
| 4 | randomised trials | serious | not serious | not serious | serious^a^ | none | 254/283 (89.8%) | 207/283 (73.1%) | **OR 3.32** (2.71 to 4.07) | **17 more per 100** (from 15 more to 19 more) | ⨁⨁◯◯ Low | IMPORTANT |
| **abdominal pain relief** | | | | | | | | | | | | |
| 5 | randomised trials | serious | not serious | not serious | serious^a^ | none | 306 | 306 | - | SMD **1.72 SD lower** (2.23 lower to 1.21 lower) | ⨁⨁◯◯ Low | IMPORTANT |
| **Abdominal pain relief_severe** | | | | | | | | | | | | |
| 3 | randomised trials | serious | not serious | not serious | serious^a^ | none | 230 | 230 | - | MD **1.68 lower** (1.86 lower to 1.5 lower) | ⨁⨁◯◯ Low | IMPORTANT |
| **Multiple Organ Disfunction Syndrome** | | | | | | | | | | | | |
| 3 | randomised trials | not serious | not serious | not serious | serious^a^ | none | 14/246 (5.7%) | 33/243 (13.6%) | **OR 0.39** (0.11 to 1.40) | **8 fewer per 100** (from 12 fewer to 4 more) | ⨁⨁⨁◯ Moderate | CRITICAL |
| **ARDS** | | | | | | | | | | | | |
| 3 | randomised trials | not serious | not serious | not serious | serious^a^ | none | 17/127 (13.4%) | 47/129 (36.4%) | **OR 0.27** (0.05 to 1.54) | **23 fewer per 100** (from 34 fewer to 10 more) | ⨁⨁⨁◯ Moderate | CRITICAL |
| **Shock** | | | | | | | | | | | | |
| 3 | randomised trials | not serious | not serious | not serious | serious^a^ | none | 28/127 (22.0%) | 47/129 (36.4%) | **OR 0.46** (0.07 to 2.94) | **16 fewer per 100** (from 33 fewer to 26 more) | ⨁⨁⨁◯ Moderate | CRITICAL |
| **AKI** | | | | | | | | | | | | |
| 3 | randomised trials | not serious | not serious | not serious | serious^a^ | none | 17/127 (13.4%) | 36/129 (27.9%) | **OR 0.29** (0.02 to 4.10) | **18 fewer per 100** (from 27 fewer to 33 more) | ⨁⨁⨁◯ Moderate | CRITICAL |

Table 11S. Summary of findings: Grading of Recommendations, Assessment, Development and Evaluations (GRADE) framework (CI: confidence interval; MD: mean difference; OR: odds ratio; SMD: standardised mean difference)

#### Explanations

a. small sample size
